# Two sources of task prioritization: The interplay of effector-based and task order-based capacity allocation in the PRP paradigm

**DOI:** 10.3758/s13414-020-02071-6

**Published:** 2020-06-12

**Authors:** Mareike A. Hoffmann, Aleks Pieczykolan, Iring Koch, Lynn Huestegge

**Affiliations:** 1 Institute of Psychology, University of Würzburg, Röntgenring 11, 97070 Würzburg, Germany; 2 Human Technology Center, RWTH Aachen University, Aachen, Germany; 3 Institute of Psychology, RWTH Aachen University, Aachen, Germany

**Keywords:** Cognitive and attentional control, Dual-task performance, Dual task procedures (PRP)

## Abstract

When processing of two tasks overlaps, performance is known to suffer. In the well-established psychological refractory period (PRP) paradigm, tasks are triggered by two stimuli with a short temporal delay (stimulus onset asynchrony; SOA), thereby allowing control of the degree of task overlap. A decrease of the SOA reliably yields longer RTs of the task associated with the second stimulus (Task 2) while performance in the other task (Task 1) remains largely unaffected. This Task 2-specific SOA effect is usually interpreted in terms of central capacity limitations. Particularly, it has been assumed that response selection in Task 2 is delayed due to the allocation of less capacity until this process has been completed in Task 1. Recently, another important factor determining task prioritization has been proposed—namely, the particular effector systems associated with tasks. Here, we study both sources of task prioritization simultaneously by systematically combining three different effector systems (pairwise combinations of oculomotor, vocal, and manual responses) in the PRP paradigm. Specifically, we asked whether task order-based task prioritization (SOA effect) is modulated as a function of Task 2 effector system. The results indicate a modulation of SOA effects when the same (oculomotor) Task 1 is combined with a vocal versus a manual Task 2. This is incompatible with the assumption that SOA effects are solely determined by Task 1 response selection duration. Instead, they support the view that dual-task processing bottlenecks are resolved by establishing a capacity allocation scheme fed by multiple input factors, including attentional weights associated with particular effector systems.

## Introduction

In everyday life, we are often confronted with situations in which we have to perform more than one task at a time. Typically, performance (in at least one of the tasks) suffers in such situations when compared with executing only one task in isolation (see Koch, Poljac, Müller, & Kiesel, 2018; Pashler, 1994, for reviews). The aim of the present study was to systematically look at two factors that determine the priority with which tasks are processed in such dual-task situations—namely, task order and the particular effector systems associated with tasks. Therefore, we combined tasks requiring oculomotor, vocal, and manual responses in pairwise combinations initiated with a short temporal delay in order to answer the question of whether task order-based task prioritization can be modulated by Task 2 effector system.

## Empirical approaches to dual tasking

Several methodological approaches have been established to address cognitive mechanisms underlying dual-task control. For example, in continuous task paradigms, participants are trained to perform two or more tasks continuously over a longer time span (e.g., Peterson, 1969; Spelke, Hirst, & Neisser, 1976; see also Künstler et al., 2018). Other research paradigms aimed at maximizing control over stimulus conditions and response execution by involving clearly defined sets of stimuli and responses with discrete onsets.

One example is the *simultaneous (stimulus) onset paradigm,* in which two stimuli (each requiring a distinct single response) are presented at exactly the same time. Performance is then compared between single-task versus dual-task conditions (e.g., Hoffmann, Pieczykolan, Koch, & Huestegge, 2019; Hoffmann, Westermann, Pieczykolan, & Huestegge, in press; Huestegge & Koch, 2013; Pieczykolan & Huestegge, 2014, 2017, 2018; Schumacher et al., 2001; Stelzel, Schumacher, Schubert, & D’Esposito, 2006). One disadvantage of this paradigm is that single-task and dual-task conditions differ with respect not only to the degree to which tasks are performed simultaneously but also whether one or two task representations need to be kept active during a particular trial. Thereby, in this paradigm it is difficult to exactly pinpoint the sources of any dual-task performance decrements (e.g., whether dual-task costs are caused by the actual motor demands in both tasks or whether mere preparation in terms of active mental representations of the two competing task sets is sufficient to yield performance decrements).

As a consequence, the probably most prominent paradigm to study dual-task processes is the *psychological refractory period* (PRP) paradigm, which involves the execution of two tasks in each trial, but with varying temporal overlap. Specifically, stimuli are presented with a short, variable temporal interval (stimulus onset asynchrony; SOA), which allows the researcher to systematically manipulate the degree of temporal overlap between cognitive requirements of the two tasks (Telford, 1931; Welford, 1952; see also Bratzke et al., 2008; Hirsch, Declerck, & Koch, 2015; Janczyk, Augst, & Kunde, 2014; Kunde, Wirth, & Janczyk, 2018; Pashler, 1984, 1994; Strobach, Becker, Schubert, & Kühn, 2015). A well-established finding is that a decrease of SOA yields an increase in reaction times (RTs) of Task 2 (i.e., the task in which the stimulus was presented second and in which the response is usually executed second), whereas Task 1 RTs typically remain unaffected by SOA manipulations. This pattern of SOA effects is typically referred to as the PRP effect (e.g., Bratzke et al., 2008; Fagot & Pashler, 1992; Janczyk & Kunde, 2010; Pashler, 1984; see also Pashler, 1994). Note that the term “PRP effect” already implies a priori the existence of a refractory period, in which no parallel processing can take place (cf. also Navon & Miller, 2002). As we want to avoid such theoretical connotations regarding the present study, we will simply refer to this predicted pattern as the “SOA effect.”

## The response selection bottleneck model

Pashler (1994) interpreted this SOA effect in dual-task performance by assuming that the central processing stages of the two tasks (i.e., the stage at which the task-appropriate response has to be selected) can only occur serially (response selection bottleneck [RSB] model), whereas stimulus processing and response execution of one task were assumed to be processed in parallel to any stage of the other task. This central bottleneck is conceptualized in an all-or-none fashion based on task order: Response selection in Task 2 cannot proceed until response selection in Task 1 is finished. This interruption of Task 2 processing has also been referred to as “slack time” (Schweickert, 1978). Note that according to this view, the SOA effect (in Task 2 RTs) is solely determined by the duration of response selection in Task 1. The main reason for the bottleneck-based prioritization of Task 1 over Task 2 is that stimulus processing in Task 1 is usually finished first (due to SOA), and thereby Task 1 arrives earlier at the central (response selection) stage.

Interestingly, an SOA effect was also demonstrated using different effector systems, including oculomotor responses (e.g., Pashler, Carrier, & Hoffman, 1993). Later, Bratzke et al. (2008) additionally demonstrated the stability of the SOA effect when the effector systems were reversed across tasks. Note, however, that this study did not explicitly focus on a comparison of SOA effect size as a function of effector system reversal across the two tasks.

## Parallel central processing models

However, the assumption of strictly serial central processing of response-related task features has been challenged on various levels (see Pieczykolan & Huestegge, 2019; see also Koch et al., 2018, for a recent review). Hommel (1998), for example, proposed the presence of partially parallel central processing of response-related features across tasks. Particularly, his data suggested that response selection of Task 1 is affected by response-related features of Task 2. Specifically, Task 1 performance was influenced by the response–response compatibility relation between the two tasks (as well as by the compatibility between the secondary response and the primary stimulus), a phenomenon referred to as backward crosstalk/compatibility effect (Hommel, 1998; see also Durst & Janczyk, 2018, 2019; Hommel & Eglau, 2002; Huestegge, Pieczykolan, & Janczyk, 2018; Janczyk, Pfister, Hommel, & Kunde, 2014; Janczyk & Huestegge, 2017; Janczyk, Renas, & Durst, 2018; Renas, Durst, & Janczyk, 2018). This suggests that at least some aspect of response-related central processing (e.g., response activation) can occur in parallel.

Moreover, there are also theories that go a step further and assume that completely parallel response selection across tasks is principally possible, and that during dual tasking, limited cognitive capacity is continuously shared among (and differentially allocated to) tasks (e.g., based on strategic reasons; Meyer & Kieras, 1997; Navon & Miller, 2002; Tombu & Jolicœur, 2003). In fact, these models make predictions quite similar to a serial response selection account. For example, they assume that a major portion of capacity is allocated to Task 1 response selection first until capacity is later shifted (in either a gradual or step-wise manner) to complete Task 2 response selection. Thus, instead of an “inflexible”, all-or-none allocation mechanism (as suggested by the term “refractory” in the term “psychological refractory period effect”) based on subtask order, these more recent theories allow for flexible, graded capacity sharing that can be modulated by particular task demands or instructions (see Fischer & Plessow, 2015, for an overview; Logan & Gordon, 2001, for a discussion; cf. also Huestegge & Koch, 2010, for a crossmodal crosstalk model).

Consequently, Navon and Miller (2002) as well as Tombu and Jolicœur (2003) argued that central capacity sharing models can yield the same predictions as a structural all-or-none bottleneck model, and that the latter can be seen as a special variant of capacity sharing. Evidence for flexible capacity allocation comes, for example, from a study by Miller, Ulrich, and Rolke (2009), who observed more parallel processing in conditions where short SOAs were particularly frequent. Taken together, capacity sharing models interpret SOA effects in terms of a reflection of a particular capacity allocation scheme (usually prioritizing Task 1), which can, however, principally be modulated by task characteristics.

## The role of effector systems in dual tasking

A different line of studies recently identified another potential factor influencing capacity allocation in multiple (simultaneous) action control situations—namely, the particular effector systems associated with the tasks at hand. A study by Huestegge and Koch (2013) on single versus dual actions triggered by single stimuli demonstrated evidence for flexible, effector-based capacity allocation between simultaneously triggered actions in different effector systems as indexed by the size of corresponding dual-action costs (defined as the RT difference between single-action and dual-action performance). More specifically, the relative size of dual-action costs suggested a prioritization of oculomotor responses (smallest dual-action costs) over vocal responses, and of vocal over manual responses (largest dual-action costs).

Recently, we demonstrated that similar effects can also be observed in another dual-task setting—namely, in the simultaneous onset paradigm involving two stimuli that independently triggered two responses (Hoffmann et al., 2019). In this particular study, we replicated the ordinal prioritization pattern referred to above (additionally including foot responses, which were prioritized over both vocal and manual responses).

In the dual-task context, these results cannot be explained by any “first come, first served” mechanism, since the effector-based prioritization (i.e., the size of dual-task costs) was not ranked in accordance with absolute task RT levels (cf. also Pieczykolan & Huestegge, 2014, 2018). Note that such a “first come, first served” mechanism is not only a part of basic RSB models of response scheduling, but also (at least implicitly) of more recent flexible capacity scheduling models. That is, even without assuming strictly serial response selection, flexible capacity sharing models also need to provide an answer to the question of how capacity is allocated among tasks, which is usually referred to stimulus sequence (or task processing speed; see, e.g., Logan & Gordon, 2001).

## Factors affecting capacity allocation

The findings described above once again highlight that the observation of response selection queuing in PRP studies is a rather special case of capacity allocation that is brought about by sequential stimulus presentation. However, several other factors were proposed that influence capacity scheduling. For example, there is already evidence that factors such as SOA distribution (e.g., Miller et al., 2009), the proportion of congruent (vs. incongruent) trials (e.g., Fischer, Gottschalk, & Dreisbach, 2014) or instructions (e.g., Lehle & Hübner, 2009) can influence how response selection capacity is shared.

Another form of capacity allocation has been discussed in the context of superordinate processes responsible for implementing task order in the form of task order sets (cf. De Jong, 1995; Hirsch, Nolden, & Koch, 2017; Hirsch, Nolden, Philipp, & Koch, 2018; Kübler, Reimer, Strobach, & Schubert, 2018; Luria & Meiran, 2003). Specifically, it is assumed that the two subtasks are not represented independently, but grouped as one single higher-order task that integrated both the individual subtask task sets and task order information. This assumption is, for instance, supported by the observation of task set switch costs (cf. Hirsch et al., 2017; Hirsch et al., 2018) as well as specific costs yielded by task order alternations (cf. e.g., Kübler et al., 2018; Luria & Meiran, 2003) or recent studies on task order control with endogenously prepared and predictable task order information (Strobach, Kübler, & Schubert, 2019).

Among all these relevant sources of influence for capacity scheduling reviewed so far, the present study is mainly focused on effector systems as highly influential factors that determine weighting mechanisms when allocating capacity to the individual response selection processes. Specifically, during parallel processing of tasks executed with different effector systems, we assume that effector-related characteristics become already functionally effective (i.e., anticipated) at a relatively early stage of task processing. Based on this, capacity is shared unevenly among tasks in that more capacity is shifted towards tasks associated with effector systems that are located higher up on an ordinal hierarchy (e.g., prioritization of oculomotor tasks over tasks involving other effector systems; cf. Hoffmann et al., 2019).

## The interplay of task order and effector systems

Therefore, both task order-related processes relevant for typical SOA effects in the PRP paradigm and effector system-related prioritization represent two sources of influence that evidently affect task prioritization in terms of capacity allocation in dual-task control. So far, however, the interplay between these two sources of task prioritization remained elusive. In particular, flexible capacity sharing accounts, which embrace the view that SOA effects are a reflection of flexible and parallel central capacity allocation, principally allow for modulations of the SOA effect on Task 2 performance. These modulations might come, for example, from effector systems associated with the tasks. Previous dual-task studies that focused on SOA effects using different effector systems did not explicitly tackle this potential modulation of the SOA effect under otherwise controlled conditions (e.g., Bratzke et al., 2008; Fagot & Pashler, 1992; Hibberd, Jamson, & Carsten, 2010; Janczyk & Kunde, 2010).

Two outcomes are conceivable: On the one hand, typical task order-based effects such as the SOA effect might be so strong that the corresponding allocation policy is immune to any effects related to effector systems. Here, we refer to the resulting temporal sequence of assigning major portions of capacity to the two tasks at the level of response selection. This would be plausible under the assumption that effector system information comes into play relatively late during task processing (e.g., only at the last stage of response execution-related processing). However, on the other hand, it is also possible that participants anticipate specific characteristics of the effector systems associated with the two tasks (e.g., anticipated proximal sensory response effects; cf. e.g., Pfister, Janczyk, Gressmann, Fournier, & Kunde, 2014; Pfister & Kunde, 2013) so that effector system prioritization mechanisms might modulate the capacity allocation scheme. The latter assumption would predict that the SOA effect on Task 2 performance in the context of the same Task 1 might vary as a function of the effector system associated with Task 2.

## The present study

To address this question, we combined oculomotor, vocal, and manual responses in three pairwise combination groups in a PRP setup involving counterbalanced auditory and visual stimuli and spatial left/right responses for the two tasks. Within these combination groups, we manipulated task order so that each effector system served as Task 1 or as Task 2 in different blocks.

We generally expected to observe higher Task 2 RTs under short SOAs than under long SOAs for all effector systems (i.e., typical influence of SOA on RT). Note that we did not focus on comparing SOA effects as a function of effector system order *within* each pairwise group of effector systems. Any significant effects of such a comparison would be confounded by the fact of a different effector system in Task 1, and could therefore not conclusively demonstrate prioritization processes among effector systems. For example, differences in the duration of an effector-specific response selection stage between the two Task 1s could also lead to asymmetrical SOA effects without providing clear evidence for an influence of Task 2 effector system. Instead, we compared SOA effects on Task 2 performance *across* pairwise groups—specifically, in dual-task settings with different Task 2 effector systems, but constant Task 1 effector systems (e.g., comparison of vocal and manual Task 2 performance that are combined with the same oculomotor Task 1).

If the SOA effect in Task 2 is mainly determined by Task 1 response selection duration (e.g., as one would expect within a classic all-or-none RSB framework), the SOA effect observed in Task 2 RTs in the context of the same Task 1 should be independent of the effector system associated with Task 2. However, if the SOA effect reflects principally flexible capacity allocation (prioritization of Task 1), and if effector systems impact on the capacity allocation scheme in dual tasking, then this allocation (reflected in the size of the SOA effect) could be modulated by the effector system associated with Task 2. Specifically, the SOA effect (as a marker of the extent to which Task 1 is prioritized over Task 2) could be smaller when Task 2 involves responses executed by effector systems with a higher priority within the ordinal effector-based prioritization pattern.

Therefore, we compared Task 2 performance as a function of the effector system of Task 2 while the effector system of Task 1 was held constant. This resulted in three separate mixed comparisons with the within-subject factor SOA and the between-subject factor group (ultimately referring to Task 2 effector system). More specifically, we compared the influence of SOA on Task 2 performance for vocal versus manual responses after an oculomotor Task 1, on oculomotor versus vocal Task 2 performance after a manual Task 1, and on oculomotor versus manual Task 2 performance after a vocal Task 1 (see Table 1 for further illustration of this logic).

**Table 1:** Overview of the three main comparisons, highlighting which effector system is associated with Task 1 (T1) and Task 2 (T2), respectively

	Constant T1	Conditions to be compared		
Comparison 1	Oculomotor T1	Vocal T2	vs.	Manual T2
		(of oculomotor–vocal group)		(of oculomotor–manual group)
Comparison 2	Manual T1	Oculomotor T2	vs.	Vocal T2
		(of oculomotor–manual group)		(of vocal–manual group)
Comparison 3	Vocal T1	Oculomotor T2 vs.	vs.	Manual T2
		(of oculomotor–vocal group)		(of vocal–manual group)

*Note*. Comparisons imply between-subject comparisons. Task order within each effector system combination pairing was manipulated within subjects

## Method

### Participants

We tested 24 participants in each of the three pairwise combination groups of oculomotor, vocal, and manual left/right tasks, resulting in a total of 72 participants. Across all three groups, 14 participants had to be excluded because their data contained too many invalid or erroneous (above a criterion of 33%) trials, or due to technical issues during data collection (three participants). We recollected these data (14 new participants) to ensure full counterbalancing and constant group size. Twenty-two out of the final 72 participants were male, and the mean age was 24.3 years (*SD* = 6.6). All were naïve regarding the purpose of the study and gave informed consent. Participants were recruited from the local university’s student panel and received monetary reward or course credit for participation. They were all right-handed and had normal or corrected-to-normal hearing and vision.

### Apparatus and stimuli

Participants were seated approx. 67 cm in front of a 21-inch cathode ray tube screen. Spatial resolution was 1,024 × 768 pixels, with a temporal resolution of 100 Hz. An eye tracker sampling eye movements at 1000 Hz (EyeLink 1000, SR Research Mississauga, Ontario, Canada) was used to register saccade latencies and direction (right eye) in conditions requiring oculomotor responses, and to control for unintended saccades in the vocal–manual combination group (in which oculomotor responses were not required). Head movements were minimized by using a chin rest. When manual responses were required, participants should operate the left and right arrow key on a standard (German) QWERTZ keyboard unimanually with their right index finger, using the arrow down key as a home key. Vocal responses were registered by a microphone (Sennheiser e 835-S) in front of them. The software Experiment Builder (Version 2.1.140, SR Research) was used to run the experiment and to log the event times of oculomotor, manual, and vocal responses. The latter was measured by means of the integrated voice key function of the programming software.

Auditory stimuli consisted of 1000 Hz sinusoidal tones presented either to the left or right ear via supra-aural headphones. Visual stimuli consisted of easily distinguishable green arrows presented 0.43° above the fixation cross pointing either to the left or to the right (“<” or “>”; size = 0.86°). Moreover, throughout each block, a green fixation cross (size = 0.43° of visual angle) at the center and two green rectangular squares at an eccentricity of 8.5 degrees of visual angle (size = 0.43° each) to the left and right of the central fixation cross remained present on the screen (black background). These green rectangular squares served as targets for oculomotor responses (when required) but were also presented in the vocal–manual group to keep the visual input constant and to ensure comparability among groups and conditions.

### Procedure

At the beginning of the experiment participants received instructions both verbally by a research assistant as well as visually on an instruction screen. The experiment consisted of four different block types. These block types were based on two task orders (e.g., vocal response first, manual response second vs. manual response first, vocal response second in the vocal–manual combination group), and, for counterbalancing reasons, two stimulus modalities and thus two stimulus to response (S–R) modality mappings. Note that in all three combination groups both possible assignments of stimulus modalities to effector systems were implemented (e.g., visual stimuli combined with manual responses and auditory stimuli combined with vocal responses; i.e., VM–AV, vs. the opposite pairing, VV–AM, VO–AM vs. VM–AO, and VO–AV vs. VV–AO) to control for any influence of the assignment of stimulus modality and effector systems. In total, there were eight blocks (64 trials each) with a total duration of approximately 45 minutes. The S–R modality mapping stayed consistent for the first half of the experiment and was changed after the first half of the experiment (order of S–R modality mappings was counterbalanced across participants). The sequence of the different task-order blocks was also counterbalanced across participants (but kept constant for each individual).

Stimuli were always presented in the order in which participants were required to respond to them. For example, in the “manual first” condition of the vocal–manual combination group, participants in the VM–AV mapping condition always saw the visual stimulus prior to hearing the auditory stimulus. Each individual block started with an instruction regarding stimulus order, corresponding required response order, and the particular S–R modality mapping.

Instructions were followed by a three-point horizontal calibration routine. Both stimuli (visual arrow and auditory sinusoidal tone) were presented for 80 ms with a variable SOA of 50, 200, 400, or 800 ms. All directional combinations of both stimuli (arrow pointing to left or right and auditory signal on the left or right ear) occurred equally often under each experimental condition in randomized order.

Participants were instructed to focus on the central fixation cross throughout each block in the vocal–manual combination group, in which oculomotor responses were not required. In groups requiring an oculomotor response (oculomotor–vocal combination group and oculomotor–manual combination group), participants were instructed to execute a saccade to either the left or the right rectangular target square (both were presented at constant eccentricity from the fixation cross) and to return with their gaze to the fixation cross afterwards. When manual responses were required, participants were asked to position their right index finger loosely (without pressing the key) on the *arrow down* key on the keyboard, which was located centrally between the two response keys (*arrow right* and *arrow left*). Participants were instructed to return to the central key position after response execution. This was done to increase comparability to oculomotor responses that also consist of a movement from a starting position to a target position. Vocal responses consisted of uttering the words “links” or “rechts” (German for “left”/“right”). Responses should always be spatially congruent to the respective stimuli.

The first of the two stimuli were presented with a fixed interstimulus interval (ISI) of 3,000 ms between two consecutive trials. If participants responded in the wrong order (as defined by instructions and stimulus order), or if they responded with one of the two effector systems before the corresponding stimulus had been presented (e.g., execution of a saccade prior to onset of the visual stimulus in the VO–AM S–R modality mapping), an error feedback was presented (300 ms, either “error” or “to soon” plus an additional reminder of the respective task requirements, e.g., “key press first” or “saccade first” in the context of a “wrong order” feedback and “pay attention to the tone/arrow” in the context of premature responses).

### Design

The within-subjects factor was SOA (50 ms, 200 ms, 400 ms, 800 ms) and the between-subjects factor was group (representing the effector system of Task 2: oculomotor, vocal, or manual, combined with the respective other effector systems in Task 1) in a mixed design. Note that we controlled for the influence of stimulus modality of Task 2 by instantiating both S–R modality mapping conditions (in counterbalanced order). For the sake of conciseness, we report results averaged over the two S–R modality mapping conditions, but we additionally provide results of separate analyses for all S–R modality mappings in Tables 2 and 3 in the Appendix. RTs and error rates (ERs) served as dependent variables. Crucial comparisons were those related to the interaction of SOA and group for the same Task 1 effector system condition. That is, in three (overlapping), pairwise mixed 4 (SOA) × 2 (group; i.e., Task 2 effector system) analyses of variance (ANOVAs), we compared SOA effects on Task 2 performance in the oculomotor–vocal versus oculomotor–manual group when the oculomotor response was associated with Task 1 (Analysis 1), on Task 2 performance in the oculomotor–manual versus vocal–manual group when the manual response was associated with Task 1 (Analysis 2), and on Task 2 performance in the oculomotor–vocal versus vocal–manual group when the vocal response was associated with Task 1 (Analysis 3; see Table 1 for a further illustration of this logic).

## Results

In case of sphericity violations, Greenhouse–Geisser corrected degrees of freedom and statistics are presented in all analyses.

### Data treatment

Of all trials, 8.5% were excluded from further analysis because they contained an omission error regarding at least one of the required responses. Further, 2.5% of trials included responses executed in the wrong order, and another 1.4% were excluded because an unwarranted saccade was registered (prior to the actually required responses) in conditions that did not require saccades. Note that omission errors were not considered in the error analysis because of effector-specific baseline differences in the occurrence of such omissions—for example, based on sensitivity differences of the devices that capture the various responses in the different effector systems.

Next, responses given within the first 50 ms after stimulus onset were discarded (further 0.3%), for example, to ensure the exclusion of voice key artefacts. Further, outliers were defined as responses slower or faster plus/minus two standard deviations from the individuals mean in each condition. These were also excluded (6.8%). In 6.0% of the remaining valid trials, a directional error (e.g., right instead of left) in at least one of the two responses was made. Only valid and correct trials were considered in the RT analyses.

### Reaction times

**Fig. 1:**
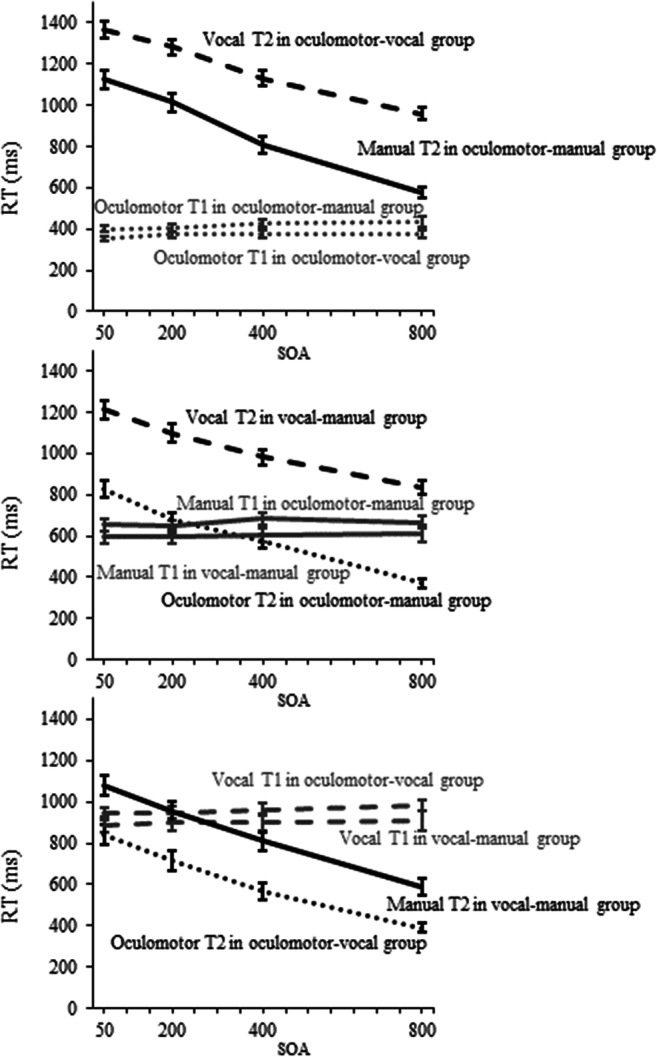
RTs for Task 1 and Task 2 for the three main comparisons. Compared conditions (i.e., those involving the same Task 1, but different Task 2 effector systems) are presented within one panel: vocal versus manual Task 2 with constant oculomotor Task 1 in the upper panel, oculomotor versus vocal Task 2 with constant manual Task 1 in the middle panel, and oculomotor versus manual Task 2 with constant vocal Task 1 at the bottom. Error bars represent (unadjusted) standard errors of the individual means (*SEM*). SOA = stimulus onset asynchrony

Figure 1 shows that typical SOA effects (in terms of an increase of Task 2 RT with decreasing SOA) were observed throughout all groups and conditions. This indicates a general prioritization of Task 1 (i.e., due to instructed task order), in line with our predictions, and represents a necessary prerequisite for any search for modulations of this effect by Task 2 effector system.

As outlined above, we were interested in differences between the SOA effects for a constant Task 1 (in terms of its associated effector system and controlled for stimulus modality) as a function of different effector systems in Task 2 (i.e., for the two lines representing Task 2 performance within each graphs in Fig. 1). This resulted in three 2 × 4 ANOVAs, with the between-subjects independent variable group (Task 2 effector system) and the within-subjects independent variable SOA.^1^

For the comparison of vocal and manual Task 2 responses after an oculomotor response in Task 1 (see top panel of Fig. 1), we observed a significant main effect of group: Manual responses (881 ms) were overall faster than vocal responses (1,184 ms), *F*(1, 46) = 33.65, *p* < .001, η_p_^2^ = .42. The main effect of SOA, representing an increase of RTs with decreasing SOAs (768 ms vs. 970 ms vs. 1,147 ms vs. 1,245 ms), *F*(1.63, 75.14) = 554.32, *p* < .001, η_p_^2^ = .92, was also significant. Crucially, we observed a significant interaction of group and SOA, showing a stronger SOA effect for manual (RT difference between shortest and longest SOA: 547 ms) than for vocal responses (RT difference between shortest and longest SOA: 409 ms), *F*(1.63, 75.14) = 11.78, *p* < .001, η_p_^2^ = .20. Additionally, as can be seen in Table 2 in the Appendix, this effect was observed in each of the two individual S–R modality mapping conditions (SOA effect: 514 ms vs. 349 ms for visually triggered Task 2, and 590 ms vs. 488 ms for auditorily triggered Task 2).

For the comparison of oculomotor and vocal Task 2 RTs following a manual response in Task 1 (see middle panel of Fig. 1), we observed that oculomotor responses (613 ms) were significantly faster than vocal responses (1,031 ms), *F*(1, 46) = 70.89, *p* < .001, η_p_^2^ = .61. Task 2 RTs increased with decreasing SOAs (603 ms; 776 ms; 889 ms; 1,019 ms), *F*(1.59, 73.17) = 376.80, *p* < .001, η_p_^2^ = .89. The interaction of group and SOA missed the critical alpha level, *F*(1.59, 73.17) = 3.21, *p* = .057, η_p_^2^ = .07. However, it should be noted that the numerical trend in this comparison was in the direction of a slightly stronger SOA effect on oculomotor (RT difference between shortest and longest SOA: 455 ms) than on vocal responses (RT difference between shortest and longest SOA: 377 ms).

In comparison of oculomotor and manual Task 2 RTs following a vocal Task 1 (see bottom panel of Fig. 1), the ANOVA revealed overall faster oculomotor (627 ms) than manual responses (857 ms), *F*(1, 46) = 14.86, *p* < .001, η_p_^2^ = .24, and increasing Task 2 RTs with decreasing SOAs (488 ms, 686 ms, 835 ms, 960 ms), *F*(1.48, 68.09) = 556.08, *p* < .001, η_p_^2^ = .29. There was no significant interaction between group and SOA, *F*(1.48, 68.09) = 1.60, *p* = .213, η_p_^2^ = .03.

Finally, we conducted two further analyses to rule out two potential alternative explanations of the data. Specifically, under the assumption of parallel capacity sharing between tasks the observed difference between vocal and manual SOA effects (after an oculomotor Task 1) might have also been caused by different durations of an effector-specific (vocal vs. manual) response selection process. If response selection for vocal responses generally takes less time than that of manual responses, this could also lead to a more pronounced manual SOA effect without necessarily indicating any prioritization (i.e., merely due to the shorter time span during which capacity had to be shared). Then, however, this should also hold true for vocal and manual Task 1 responses combined with an oculomotor Task 2. Therefore, to ensure that the observed difference between vocal versus manual SOA effects was not solely based on such an effector-specific response selection duration, we additionally compared the oculomotor SOA effects as a function of Task 1 effector system. That is, in addition to the rationale depicted in Table 1, we now also compared Task 2 performance of the oculomotor–vocal versus oculomotor–manual combination groups when Task 2 was associated with the oculomotor effector system and Task 1 with the vocal versus manual system, respectively. The corresponding 4 (SOA) × 2 (group, i.e., now referring to different Task 1 effector systems) ANOVA revealed a significant effect of SOA, *F*(1.50, 69.18) = 372.01, *p* < .001, η_p_^2^ = .89, but, crucially, neither a significant effect of group, *F*(1, 46) = 0.08, *p* = .777, η_p_^2^ = .00, nor a significant interaction, *F*(1.50, 69.18) = 0.71, *p* = .547, η_p_^2^ = .02. Thus, we can rule out that the differences between vocal and manual SOA effects could be explained in terms of different durations of effector-specific vocal versus manual response selection processes.

Second, one could also argue that different effector-system combinations might go hand in hand with a different amount of cross-task interference based on spatial response–response incompatibility. For example, one might assume that more interference might occur between an oculomotor response to the “right” in Task 1 and an incompatible manual “left” key press in Task 2 than with a merely *conceptually* incompatible “left” vocal response in Task 2 (as oculomotor and manual responses are both *physically* spatial in nature). If so, it would not be surprising when asymmetric compatibility effects interact with SOA, as one would generally expect more interference at short SOAs. To rule out this alternative explanation for our finding that a stronger SOA effect occurred for manual than for vocal responses, we additionally compared spatial compatibility effects in manual versus vocal Task 2 performance after a (compatible vs. incompatible) oculomotor Task 1 and their interaction with SOA by conducting a corresponding 2 × 2 × 4 ANOVA. Results revealed that response–response compatibility indeed affected Task 2 RTs, *F*(1, 46) = 12.31, *p* = .001, η_p_^2^ = .21, as there were faster Task 2 responses after a compatible (1,023 ms) than after an incompatible Task 1 (1,045 ms). Furthermore, this compatibility effect interacted with SOA, *F*(3, 138) = 5.83, *p* = .001, η_p_^2^ = .11, in that it decreased with increasing SOAs (39 ms, 38 ms, 9 ms, 0 ms from short to long SOA). Crucially however, the ANOVA revealed neither an interaction of compatibility and Task 2 effector system, *F*(1, 46) = 0.27, *p* = .604, η_p_^2^ = .01, nor a three-way interaction of compatibility, Task 2 effector system, and SOA, *F*(3, 138) = 0.56, *p* = .645, η_p_^2^ = .01, thereby ruling out asymmetric compatibility effects as a potential alternative explanation for the observed asymmetries in the SOA effect between vocal and manual Task 2 responses. In addition, note that our main result (smaller vocal than manual SOA effect) could be observed for both compatible and incompatible trials in corresponding separate analyses (see Table 4 in the Appendix).

### Error rates

We conducted the same three ANOVAs for Task 2 error rates (ERs), even though errors occurred relatively rarely overall (6% of valid data).

Regarding the comparison of Task 2 ERs between vocal versus manual Task 2s (after an oculomotor Task 1) ERs increased significantly with decreasing SOAs (1.5%, 2.6%, 3.1%, 5.1%), *F*(2.32, 106.89) = 12.64, *p* < .001, η_p_^2^ = .22, but did not differ significantly overall between the vocal (2.6%) versus the manual (3.3%) task, *F*(1, 46) = 0.48, *p* = .494, η_p_^2^ = .01. Unlike in RTs, there was no interaction of group and SOA regarding ERs, *F*(2.32, 106.89) = 0.69, *p* = .526, η_p_^2^ = .02.

Similarly, the comparison of vocal versus oculomotor Task 2 ERs (after a manual Task 1) revealed a significant effect of SOA (2.9%, 4.4%, 5.9%, 5.7%), *F*(3, 138) = 8.79, *p* < .001, η_p_^2^ = .16, but neither an effect of group (oculomotor: 4.9%; vocal: 4.5%), *F*(1, 46) = 0.06, *p* = .803, η_p_^2^ = .00, nor an interaction, *F*(3, 138) = 1.59, *p* = .194, η^2^ η_p_^2^_p_ = .03.

Lastly, comparing oculomotor versus manual Task 2 ERs (after a vocal Task 1) revealed a significant effect of group (indicating higher ERs in oculomotor: 4.5% than in manual responses: 1.5%), *F*(1, 46) = 18.27, *p* < .001, η_p_^2^ = .28, but no effect for SOA (2.5%, 3.9%, 5.6%, 6.9%), *F*(2.26, 104.04) = 1.77, *p* = .156, η_p_^2^ = .04, and no interaction, *F*(2.26, 104.04) = 1.36, *p* = .259, η^2^_p_ = .03.^2^

## Discussion

The aim of the present study was to test whether the effect of SOA on Task 2 performance (i.e., an increase of Task 2 RTs with decreasing SOA), which is typically interpreted as a reflection of strong initial capacity allocation to Task 1 response selection, can be modulated by the Task 2 effector system. Here, we assume that the SOA effect is regarded as indexing a flexible capacity allocation regime. Importantly, based on this reasoning, it is possible that the SOA effect can then be affected by effector systems associated with the tasks, we can expect a modulation of the SOA effect as a function of the effector system associated with Task 2 (cf. e.g., Hommel, 1998; Meyer & Kieras, 1997; Navon & Miller, 2002; Tombu & Jolicœur, 2003), in line with the ordinal prioritization pattern regarding effector systems established in prior research (Hoffmann et al., 2019; Hoffmann et al., in press; Huestegge & Koch, 2013; Pieczykolan & Huestegge, 2014).

Here, we addressed this issue by systematically combining comparable PRP tasks (always involving simple left/right decisions based on left/right stimuli) that differed with respect to their associated effector systems (oculomotor, manual, vocal). All pairwise combinations of these effector systems were implemented across three groups of participants, while effector system (or task) order was manipulated and S–R modality mapping was controlled for within each group.

### Summary of main findings

We observed the expected SOA effect (prolongation of Task 2 RTs with decreasing SOA) in all groups and conditions. The most important result here is that the SOA effect depended on the Task 2 effector system under constant Task 1 requirements (i.e., when Task 1 involved the same effector system), at least for the oculomotor Task 1. Specifically, following the oculomotor Task 1, the SOA effect was more pronounced when Task 2 involved a manual response compared with a vocal response (SOA effect, measured as the RT difference between the shortest and the longest SOA: 547 ms vs. 409 ms).

This observation of an effector system-dependent SOA effect further strengthens the idea of flexible capacity sharing among tasks and speaks against a basic all-or-none response selection bottleneck model without major additional assumptions, as according to the latter the slack time responsible for the SOA effect (which is determined by Task 1 response selection duration) should be independent of Task 2 response characteristics. Further, it shows that effector systems are an influential factor in the decision process that determines how capacity is allocated among tasks: task prioritization is based not only on task order (as posited by both all-or-none and capacity sharing explanations of SOA effects) but also on specific effector system characteristics. Note that the interpretation of this effect was not compromised by the error data, because there was no counteracting effect in the error data regarding the crucial interaction of Task 2 effector system and SOA. Also note that this finding of vocal-over-manual prioritization corroborates findings from previous studies using other multitasking settings (e.g., Hoffmann et al., 2019; Hoffmann et al., in press; Huestegge & Koch, 2013; Pieczykolan & Huestegge, 2014).

### Potential alternative explanations

At first sight, one might argue that such a modulation of the SOA effect by Task 2 effector system could also have resulted from effector system-specific (and thus unequally long) response selection processes in Task 2 (assuming the occurrence of parallel response selection processes in the first place), or from an asymmetry among Task 2 effector systems in the susceptibility to incompatible Task 1 information. However, the lack of any significant influence of Task 1 effector system on oculomotor Task 2 SOA effects and the finding of similar patterns for spatially compatible and incompatible trials render these explanations highly unlikely.

Finally, another general objection against our interpretation of the data might be to argue that the finding of smaller SOA effects for conditions involving a larger RT difference between Task 1 and Task 2 (as was the case for the vocal vs. manual Task 2 when combined with an oculomotor Task 1) should generally be expected, even based on most rudimentary response selection bottleneck models. At first sight, this might appear reasonable since large RT differences across tasks might decrease the potential for temporal overlap of response selection stages. However, it is crucial to bear in mind that we took great care in controlling stimulus-related processing duration in our relevant comparisons. Thereby, the end point of stimulus processing (which marks the crucial onset of the central bottleneck stage) is perfectly comparable, and thus there is no reason to assume a lower potential for central (response selection) stage overlap/competition for a vocal versus manual Task 2 given the same (oculomotor) Task 1. Crucially, Task 1 response selection duration (which should be the main determinant of any SOA effect in Task 2) should be perfectly comparable, and thus any basic bottleneck model would never predict differences in SOA effects based on different response-related Task 2 characteristics (i.e., differences in T2 effector system).

Consequently, our main finding can be interpreted as showing that task order-based task prioritization is affected by the effector systems involved in the tasks. Furthermore, the direction of the modulatory effect (larger SOA effect for manual Task 2) is consistent with the effector-based prioritization pattern assumed by Huestegge and Koch (2013; see also Hoffmann et al., 2019; Hoffmann et al., in press; Pieczykolan & Huestegge, 2014), where it was suggested that vocal responses are generally prioritized over manual responses despite the fact that vocal responses are usually characterized by longer overall response latencies.

### Are oculomotor responses special?

Our results show no significant differences in the SOA effect between the oculomotor versus vocal Task 2 following a manual Task 1, or between the oculomotor versus manual Task 2 following a vocal Task 1, although it should be mentioned that there was a trend towards a slightly higher oculomotor than vocal SOA effect in the former comparison. At first sight, the lack of a modulation of SOA effects in comparisons involving oculomotor Task 2 responses might appear to be at odds with the significant modulation of the SOA effect reported above (there was even a numerical trend towards oculomotor subordination instead of dominance), especially since previous research has provided strong evidence for a general prioritization of the oculomotor system over all other effector systems. However, if we take a closer look at the absolute oculomotor RT levels in comparison to manual and vocal Task 1 RTs (see Fig. 1), a reasonable explanation can be derived: Overall, oculomotor responses could be executed very quickly (about 395 ms as Task 1, compared with a Task 1 RT range of 637–936 ms for vocal and manual responses). When comparing oculomotor and vocal (or manual) Task 2 performances (after a manual or vocal Task 1, respectively), it is quite likely that under short SOAs oculomotor responses were artificially withheld until the execution of the respective Task 1 response.

Note that this effect might have been further reinforced by our decision to provide error feedback in those trials in which participants responded in a reversed order than indicated by stimulus order. In future research, it might be of interest to investigate potential oculomotor dominance effects in the PRP paradigm when violations of task order are less prominently (or not at all) fed back to the participants. For example, Bratzke and colleagues (Bratzke, Rolke, Ulrich, & Peters, 2007; Bratzke et al., 2008) repeatedly observed SOA effects even when participants were not explicitly instructed to execute responses in the order of corresponding stimulus presentation. However, such an approach could be challenging because a certain response order is a prerequisite of interpreting Task 2 RT patterns in terms of an order-based SOA effect, and the few PRP studies involving oculomotor responses to date have shown that oculomotor responses are often executed first even when the corresponding stimulus was presented second (Pashler et al., 1993; Pieczykolan & Huestegge, 2019). Thus, it might be possible that although oculomotor dominance (see Hoffmann et al., 2019; Huestegge & Koch, 2013; Pieczykolan & Huestegge, 2014) might also have an impact on cognitive processes in situations with sequential stimulation, a corresponding effect in the data may be difficult to demonstrate empirically.

### Theoretical implications

Taken together, the present results indicate that task order-based task prioritization (as typically assumed to explain SOA effects) can in fact be modulated by effector-based task prioritization. On a general level, these data are well in line with capacity sharing accounts of dual-task control (as, e.g., suggested by Tombu & Jolicœur, 2003), which postulate that task processing can also run in parallel and does not have to strictly stick to an all-or-none capacity allocation scheme as suggested by classic bottleneck accounts. In addition, our results suggest that this specific allocation scheme—indicating which capacity portion is allocated to which task—depends on several task characteristics, including effector systems and their respective prioritization weight. However, note that the present study was not designed to ultimately decide between capacity sharing and (more complex variants of) all-or-none bottleneck models but rather to explore the degree to which capacity allocation might be influenced by effector-specific factors.

Potentially, this capacity allocation mechanism could be influenced by strategic factors in order to fulfill challenging task requirements in a time-limited setting as efficiently as possible. While in conditions involving sequential stimulus onset it provides strategic advantages to shift a lion’s share of available capacity towards the task that was triggered first to get it “out of the way” and to reduce task interference, the extent of this task order-based task prioritization (as measured in the SOA effect on Task 2 RT) apparently can to some degree be adjusted as a function of more peripheral task characteristics.

The fact that certain task features which—according to traditional task processing logic—are usually regarded as “postcentral” (i.e., effector systems associated with the tasks) have such an effect on central capacity allocation policies do not seem to be consistent with basic assumptions of sequential task processing models. More specifically, these findings rather appear to support previous ideas that effector system representations are an integral part of central codes in working memory that need to be configured to eventually come up with task-appropriate action patterns. One model that considers this intuition was introduced by Huestegge and Koch (2010). Here, it was assumed that multiple-action control essentially requires the binding of task-relevant codes (e.g., spatial codes as well as codes specifying effector systems) in working memory (i.e., prior to action execution). Thus, according to this view, effector systems are an essential part of a central, complex code selection/binding process, and effector-related processing weights in such a model might be represented in terms of different connection strengths towards different effector systems. However, more research is needed to further substantiate the predictions of such a multiple action control model.

## Conclusion

In sum, we can conclude that effector system characteristics—which, on a superficial level, might be assumed to exclusively influence relatively late processing stages—evoked substantial effects on dual-task control (Huestegge, 2011; Huestegge & Adam, 2011; Huestegge & Hazeltine, 2011; Huestegge, Pieczykolan, & Koch, 2014). This highlights the importance of considering the particular effector systems involved in dual tasks when interpreting typical performance effects such as those resulting from classic SOA manipulations. Finally, the present study sheds further light on the specific mechanisms determining potential allocation regimes in capacity sharing models by indicating that several sources of influence, including task order and anticipatory processes regarding effector systems, are integrated to eventually determine a specific, demand-appropriate capacity allocation policy.

## Appendix

**Table 2.** Overview of statistical test results of six separate ANOVAs for all possible specific Task 1 conditions regarding effector system and stimulus modality with RTs of Task 2 as the dependent variable, group (i.e., effector system of Task 2) as the independent between-subjects variable, and SOA as the independent within-subjects variable

Source of variation	*df*	*F*	*p*	η_p_^2^	*df*	*F*	*p*	η_p_^2^
	Auditory–oculomotor Task 1				Visual–oculomotor Task 1			
Group (Manual vs. Vocal Task 2)	1, 46	29.70	<.001	.39	1, 46	33.97	<.001	.43
SOA	1.68, 77.30	365.44	<.001	.89	1.98, 91.15	443.22	<.001	.91
Group × SOA	1.68, 77.30	13.64	<.001	.23	1.98, 91.15	4.46	.014	.09
	Auditory–manual Task 1				Visual–manual Task 1			
Group (Oculomotor vs. Vocal Task 2)	1, 46	61.97	<.001	.57	1, 46	75.30	<.001	.62
SOA	1.59, 73.32	190.03	<.001	.81	2.19, 100.82	350.62	< .001	.88
Effector System × SOA	1.59, 73.32	1.38	.255	.03	2.19, 100.82	2.13	.119	.04
	Auditory–vocal Task 1				Visual-vocal Task 1			
Group (Oculomotor vs. Manual Task 2)	1, 46	10.81	.002	.19	1, 46	14.49	<.001	.24
SOA	2.05, 94.38	376.23	<.001	.89	1.60, 73.79	421.29	<.001	.92
Effector System × SOA	2.19, 94.38	1.90	.155	.04	1.60, 73.79	2.07	.143	.04

**Table 3.** Overview of statistical test results of six separate ANOVAs for all possible specific Task 1 conditions regarding effector system and stimulus modality with ERs in Task 2 as a dependent variable, group (i.e., effector system of Task 2) as an independent between-subjects variable, and SOA as an independent within-subjects variable

Source of variation	*df*	*F*	*p*	η_p_^2^	*df*	*F*	*p*	η_p_^2^
	Auditory–oculomotor Task 1				Visual–oculomotor Task 1			
Group (Manal vs. Vocal Task 2)	1, 46	3.04	.088	.06	1, 46	0.42	.520	.01
SOA	1.90, 87.29	10.39	<.001	.18	2.62, 120.48	2.77	.052	.06
Group × SOA	1.90, 87.29	0.10	.900	.00	2.62, 120.48	1.78	.161	.04
	Auditory–manual Task 1				Visual–manual Task 1			
Group (Oculomotor vs. Vocal Task 2)	1, 46	1.78	.189	.04	1, 46	0.29	.591	.01
SOA	2.49, 114.55	7.83	<.001	.15	2.37, 108.95	3.38	.020	.07
Effector System × SOA	2.49, 114.55	1.60	.201	.03	2.37, 108.95	0.74	.531	.02
	Auditory–vocal Task 1				Visual–vocal Task 1			
Group (Oculomotor vs. Manual Task 2)	1, 46	10.58	.002	.19	1, 46	12.70	.001	.22
SOA	2.27, 104.18	6.19	.002	.12	3, 138	5.09	.002	.10
Effector System × SOA	2.27, 104.18	0.29	.776	.01	3, 138	5.93	.001	.11

**Table 4.** Statistical test results of the supplementary analyses of Task 2 RT differences between manual versus vocal Task 2s after an oculomotor Task 1 presented separately for response–response compatible trials (left) versus response–response incompatible trials (right)

Source of variation	Compatible trials only				Incompatible trials only			
	*df*	*F*	*p*	η_p_^2^	*df*	*F*	*p*	η_p_^2^
Group (Manal vs. Vocal Task 2)	1, 46	32.15	<.001	.41	1, 46	34.18	<.001	.43
SOA	1.69, 77.94	441.67	<.001	.91	2.26, 103.77	478.43	<.001	.91
Group × SOA	1.69, 77.94	9.24	.001	.17	2.26, 103.77	10.13	<.001	.18

## Footnotes

1 Note that we also analyzed Task 1 performance as a function of Task 2 effector system. However, we observed no significant influence of Task 2 effector system, either on overall Task 1 RTs or regarding the dependency of Task 1 RTs on SOA (all *p*s > .05). This suggests that the particular effector system associated with Task 2 did not affect Task 1 processing.

2 It should be mentioned that when analyzing error rates for the two S–R modality mappings separately (see Table 3 in the Appendix), we did in fact observe a typical SOA effect when comparing *visually* triggered oculomotor and manual Task 2 ERs after an *auditory*-vocal Task 1 (2.5%, 3.9%, 5.6%, 6.9% over decreasing SOAs). This was, however, cancelled out by an unexpected significant effect for SOA in the opposite direction in *auditorily* triggered oculomotor ERs after a *visual*-vocal Task 1 (SOA 800 ms: 4.3%, SOA 400 ms: 3.7%, SOA 200 ms: 2.1%, SOA 50 ms: 0.7%). Since there was no notable alteration of the generally low manual ERs among SOAs (0.2%, 1.0%, 0.8%, 0.5%), this resulted in a significant effect of SOA overall (in an unusual pattern: 2.2%, 2.3%, 1.4%, 0.6%) as well as in an interaction of SOA and group.
